# Identification and validation of a major QTL for kernel length in bread wheat based on two F_3_ biparental populations

**DOI:** 10.1186/s12864-022-08608-3

**Published:** 2022-05-19

**Authors:** Xinlin Xie, Shuiqin Li, Hang Liu, Qiang Xu, Huaping Tang, Yang Mu, Mei Deng, Qiantao Jiang, Guoyue Chen, Pengfei Qi, Wei Li, Zhien Pu, Ahsan Habib, Yuming Wei, Youliang Zheng, Xiujin Lan, Jian Ma

**Affiliations:** 1 Triticeae Research Institute, Sichuan Agricultural University, Chengdu, 611130 China; 2 State Key Laboratory of Crop Gene Exploration and Utilization in Southwest China, Sichuan Agricultural University, Chengdu, 611130 China; 3 College of Agronomy, Sichuan Agricultural University, Chengdu, 611130 China; 4 Biotechnology and Genetic Engineering Discipline, Khulna University, Khulna, 9208 Bangladesh

**Keywords:** Kernel length, BSA-660 K SNP array, QTL identification and validation, F_3_ biparental populations

## Abstract

**Background:**

High yield and quality are essential goals of wheat (*Triticum aestivum* L.) breeding. Kernel length (KL), as a main component of kernel size, can indirectly change kernel weight and then affects yield. Identification and utilization of excellent loci in wheat genetic resources is of great significance for cultivating high yield and quality wheat. Genetic identification of loci for KL has been performed mainly through genome-wide association study in natural populations or QTL mapping based on genetic linkage map in high generation populations.

**Results:**

In this study, an F_3_ biparental population derived from the cross between an EMS mutant BLS1 selected from an EMS-induced wheat genotype LJ2135 (derived from the hybrid progeny of a spelt wheat (*T*. *spelta* L.) and a common wheat) mutant bank and a local breeding line 99E18 was used to rapidly identify loci controlling KL based on Bulked Segregant Analysis (BSA) and the wheat 660 K single-nucleotide polymorphism (SNP) array. The highest ratio of polymorphic SNPs was located on chromosome 4A. Linkage map analysis showed that 33 Kompetitive Allele Specific PCR markers were linked to the QTL for KL (*Qkl.sicau-BLE18-4A*) identified in three environments as well as the best linear unbiased prediction (BLUP) dataset. This QTL explained 10.87—19.30% of the phenotypic variation. Its effect was successfully confirmed in another F_3_ population with the two flanking markers *KASP-AX-111536305* and *KASP-AX-110174441*. Compared with previous studies and given that the of BLS1 has the genetic background of spelt wheat, the major QTL was likely a new one. A few of predicted genes related to regulation of kernel development were identified in the interval of the detected QTL.

**Conclusion:**

A major, novel and stable QTL (*Qkl.sicau-BLE18-4A*) for KL was identified and verified in two F_3_ biparental populations across three environments. Significant relationships among KL, kernel width (KW) and thousand kernel weight (TKW) were identified. Four predicted genes related to kernel growth regulation were detected in the interval of *Qkl.sicau-BLE18-4A*. Furthermore, this study laid foundation on subsequent fine mapping work and provided a possibility for breeding of elite wheat varieties.

**Supplementary Information:**

The online version contains supplementary material available at 10.1186/s12864-022-08608-3.

## Background

The annual increase rate of wheat (*Triticum aestivum* L) yield will be deficient to meet the future needs of the rapidly growing population. The decrease of genetic diversity among wheat varieties under the modern breeding mode is one of the reasons for the above phenomena [1]. Spelt wheat (*T. spelta* L.), one of the hexaploid wheats, is an archaic cereal with the primitive genomes related to bread wheat. Spelt wheat has high nutritional compositions, and its microelement content is higher and more abundant than common wheat [2, 3]. Spelt and bread wheat have the same genome (AABBDD), and their genetic distance is comparatively small, which is helpful for the production of stable cross breeds [4]. Compared with ancestral wheat species, the phenotypic variation of kernel traits in the modern germplasm pool is significantly reduced [5]. Therefore, it is of important significance to excavate new loci controlling kernel traits in wheat breeding. Kernel length (KL), as one of the main components that constitute kernel size, is a complex quantitative trait controlled by multiple genes. It can indirectly change kernel weight and ultimately affect yield [6]. Furthermore, genetic and phenotypic structures support variations in kernel size and shape. Kernel size increases gradually through changes in KW and KL, and in the later stage, changes in kernel shape are mainly realized through changes in KL [5]. For example, *TaGL3-5A* was co-located with a significant QTL for KL. Correlation analysis revealed that the *TaGL3-5A-G* allele was significantly correlated with longer KL and higher thousand kernel weight (TKW) [7]. *TaGS-D1* is associated with kernel weight and KL in common wheat [8]. Thus, it is extremely important to identify and utilize loci associated with KL in wheat gene resources for cultivating wheat with high yield and quality.

As typical quantitative traits, kernel traits are sensitive to environmental effects. The application of quantitative trait loci (QTL) provides an efficient method for studying complex quantitative traits [9]. Numerous studies related to QTL for kernel traits have been reported in wheat. For example, QTL for KL were detected on chromosomes 1B, 2B, 2D, 3B and 7B [10]. Thirteen QTL affecting KL were identified on chromosomes 2A, 2B, 2D, 3A, 6B, 7A, and 7B [11]. *TAGS-D1*, a homologous gene of *OsGS3* in rice, was located on 7DS and increased KL and kernel weight [8]. Three QTL (*QGw.nau-2D, QGw.nau-4B*, and *QGw.nau-5A*) had effects on KL, kernel width (KW) and kernel thickness (KT) of wheat [12]. A single nucleotide polymorphism (SNP) locus was found to be closely linked to a QTL associated with KL on 7AL [13]. A major QTL on 5A increased KL by increasing the length of kernel epidermis cells to achieve the purpose of enhancing kernel weight [14]. Three major QTL *QKL.sicau-2D*, *QKW.sicau-2D* and *QTKW.sicau-2D* for KL, KW and TKW were identified in the same interval on 2DS [15]. *QKL.sicau-2SY-1B*, *QKW.sicau-2SY-6D*, *QKT.sicau-2SY-2D*, and *QTKW.sicau-2SY-2D*, *QLWR.sicau-2SY-6D*, *QKS.sicau-2SY-1B/2D/6D*, and *QFFD.sicau-2SY-2D* for KL, KW, KT, TKW, kernel length–width ratio (LWR), kernel size (KS), and factor form density (FFD) were located on 1B, 2D, and 6D and formed 3 QTL clusters [16]. Eleven QTL affecting KL and KW including two major QTL *QKL.sicau-AM-3B* and *QKW.sicau-AM-4B* were identified on 3BL and 4BL [17].

According to the extreme differences of individual phenotypes in the offspring population produced by a pair of parents with related traits, two gene banks were constructed by screening and collecting DNA samples for of Bulked Segregant Analysis (BSA). This method usually provided a convenient and quick method for identifying markers for genomic regions associated with target traits [18, 19]. In previous studies, PCR-based molecular markers, such as amplified fragment length polymorphism (AFLP) and simple sequence repeats (SSR), had been generally used for gene mapping, but these markers usually take a long time to construct the map, and the density of map cannot meet the demand of fine mapping [20]. SNPs are important resource of polymorphic markers and can be used for gene mapping in the genome of any living organism. In the process of genotyping and marker-assisted selection, the wheat 660 K SNP array is an accurate, economical and dependable option [21].

To our knowledge, many reports have shown that using early generations of wheat to rapidly conduct major and stable QTL analysis combined with BSA and the wheat 660 K SNP array. For example, on the basis of BSA and the wheat 660 K SNP array in F_1_, F_2_, and F_2:3_ populations, *YrZl31* conferring stripe rust resistance was mapped on chromosome 2BL [20]. *Qyryac.nwafu-2BS,* a novel QTL for adult plant resistance to stripe rust was identified on 2B using BSA and 660 K SNP array in an F_2:3_ population [22]. The above method has been widely used to identify wheat resistance genes, but rarely applied in quantitative traits. QTL related to KL in wheat have been detected on almost each of the chromosomes, but they were usually shown as micro-effect and unstable. Most of them were detected in a single population and not verified in diverse backgrounds. Therefore, it is necessary for wheat breeding to excavate and identify major and stable QTL for KL.

In this study, we employed early generations of wheat population in combination with BSA-660 K SNP array to detect genetic differences between two pools with extreme phenotypes of KL. And we further identify and validate major and stable QTL for KL across different environments base on a linkage map.

## Materials and methods

### Plant materials

**Fig. 1:**
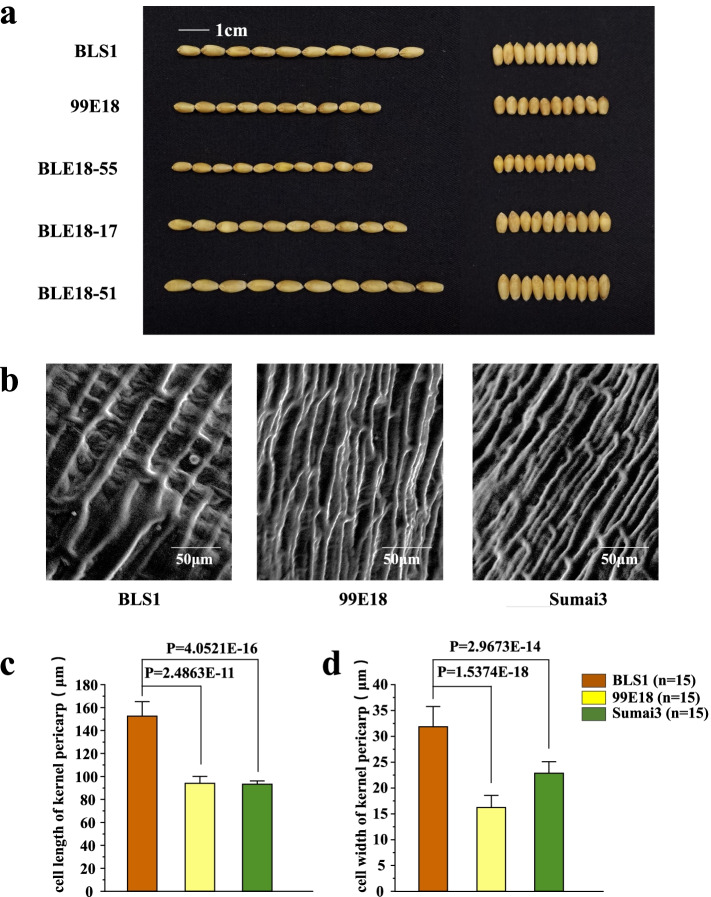
The phenotypes of kernels and pericarp cells. **a** Kernel phenotypes of the parents and partial lines in BLE18 population. Scale bar = 1 cm; **b** Scanning electron microscope observation of pericarp cells in mature kernels. Scale bar = 50 μm; **c, d.** Statistical analysis of cell length and width of kernel pericarp cells

Two F_3_ biparental populations derived from the crosses between three wheat genotypes: BLS1 and 99E18 (BLE18, the mapping population) and between BLS1 and Sumai3 (BLSM3, the validation population), comprising 237 and 178 lines, respectively, were used in this study. BLS1 is a genetically stable mutant (M5 generation) selected from an EMS-induced wheat genotype LJ2135 mutant bank. LJ2135 was a genetically stable line and is derived from the hybrid progeny of a spelt wheat (*T. spelta* L., LS5893) and a common wheat (JM6893), Given its relatively better agronomic traits including appropriate plant height and large spike, LJ2135 was selected to be a breeding parent. The KL of BLS1 can reach 8 mm under conventional cultivation conditions. The lines 99E18 and Sumai3 show shorter KL than BLS1 (Fig. 1a and Figure S1), and both have excellent disease resistance [23], especially Sumai3, which is a classic wheat variety with famous fusarium head blight resistant gene, *Fhb1* [24].

### Phenotypic evaluation

In October of 2020, the BLE18 and BLSM3 populations were grown in three different environments including Wenjiang (103°51ʹE, 30°43ʹN), Chongzhou (103°38ʹE, 30°32 ʹN), and Ya’an (103°0ʹE, 29°58ʹN) of Sichuan Province in China. Each line was planted in a single 1.5 m row with 0.3 m between rows, and 15 kernels were sown in a row with 0.1 m between kernels within a row. The field management followed the local practices of wheat production. After the harvested kernels were dried at 37 °C in an oven until a constant dry weight was obtained [25], 36 full and uniform kernels mainly from the spikelets located in the middle of the spike of different plants in each line were selected and scanned by the Epson Expression 10,000 XL flatbed color image scanner (Seiko Epson Corporation, Japan) and further analyzed by WinSEEDLE (Regent Instruments Canada Inc) for measurements of KL and KW. Cell length and width of kernel pericarp from three parents were observed and measured using the Quanta 450FEG scanning electron microscope. Different lines were characterized phenotypically in different environments. Totally, 239, 218 and 137 lines of BLE18 were measured in Wenjiang, Chongzhou, and Ya’an, respectively. Notably, some lines with poor-quality kernels especially in Ya’an were not used for further analysis, and the statistics of lines used for kernel investigation in Ya’an were listed in Table S1. Hundred kernel weight (HKW) was obtained by randomly weighting 100 kernels per line through an electronic balance with the precision of 0.01 g, and TKW calculated as 10-folds of the average HKW with three replicates.

### BSA, Wheat 660 K SNP array analysis, and KASP markers development

The combination of BSA and the wheat 660 K SNP array was performed to identify SNPs between two parents and two phenotypically contrasting pools. The 30 lines in each of two relative extreme mixing pools were selected through the following steps: (1) The phenotypic data of KL of BLE18 in three environments were arranged in a descending order. The number of 40 lines with the maximum and minimum values in the three environments were respectively taken and recorded. (2) Average values of each line from three-environments phenotypic data of KL were calculated and recorded. (3) The intersection of the numbers obtained in (1) and (2) was screened, and 30 lines with the maximum and minimum values were finally selected. The equal amounts of genomic DNA from 30 lines with the shortest KL (10 kernels per line), 30 lines with the longest KL (10 kernels per line) and two parents were extracted using the CTAB method [26]. The DNA were further used for the wheat 660 K SNP array analysis in Beijing CapitalBio Technology Co, Ltd. Two parent pools were used to exclude interference of false positive markers. For example, one marker showing polymorphic between two phenotypically contrasting pools, but not between parents will be excluded.

The polymorphic SNPs between two extreme mixing pools associated with the target QTL were converted into Kompetitive Allele Specific PCR (KASP) markers to construct linkage map. The sequences of primers were designed as per the KASP primer design manual [27], and the primer sequences are listed in Table S2. The allele-specific forward primers were designed carrying FAM (5′-GAAGGTGACCAAGTTCATGCT-3′) and HEX (5′-GAAGGTCGGAGTCAACGGATT-3′) at the 5′ end. In this study, the KASP amplification reaction was conducted in volume of 10 μl including 5 μl of 1 × SsoFast EvaGreen mix (Bio-Rad, Hercules, CA, USA), 1.4 μl of mixture forward and reverse primers, 3.1 μl of deionized water and 0.5 μl of 50–80 ng/ μl DNA. PCR cycling was performed using the following procedure: hot starting at 94 °C for 15 min, 10 touchdown cycles (94 °C for 20 s; touchdown 60 °C, drop 0.6 °C per cycle, for 60 s), and then by 25 cycles of amplification at 95 °C for 20 s, and 55 °C for 60 s.

### Genotyping and genetic map construction

A total of 33 KASP markers were developed to detect SNP polymorphism between two parents, and the effective polymorphic markers were further used to genotype BLE18 population. The results of markers’ classification were applied to construct the genetic linkage map with the Kosambi function format in JoinMap 4.0. The sequences of two flanking makers were used to blast (*E*-value of 1e-5) against the genome assembly of IWGSC RefSeq v2.1 (http://202.194.139.32/blast/blast.html) to obtain the physical locations [28]. The linkage map was drawn by MapChart 2.2.

### Data analysis and QTL mapping

Phenotypic variation, frequency distributions, and Pearson’s correlation coefficient were implemented using SPSS 22 (IBM SPSS, Armonk, NY, USA). The best linear unbiased prediction (BLUP) dataset of KL under three environments were calculated using SAS V8.0 (SAS Institute, Cary, NC, USA; https://www.sas.com). The BLUP were calculated based on the following model: *Y*_*i*_ = *X*_*i*_*f* + *a*_*i*_ + *e*_*i*_, where *f* is a fixed-effects vector, Xi is an incidence vector, *a*_*i*_ is the value of phenotype and *e*_*i*_ is the environmental deviation [29]. Based on the genetic linkage map and kernel phenotypic data in three environments as well as the BLUP, we identified the QTL for KL using IciMapping 4.1 with the Inclusive Composite Interval Mapping (ICIM) setting the LOD threshold ≥ 2.5 [30]. In addition, IciMapping 4.1 was used to analyze QTL × environment (QE) interaction, and the pre-adjusted parameters were as follows: step = 1 cM, PIN = 0.001 and LOD = 2.5. When the Percentage Variation Explained (PVE) of a given QTL was greater than 10% and can be detected repeatedly in multiple environments, it was considered as a major and stably expressed QTL. QTL was named in accordance with the International Rules of Genetic Nomenclature [16]. For example, the investigated QTL was designated as *Qkl.sicau-BLE18-4A*. In detail, ‘sicau’ means ‘Sichuan Agricultural University’; the uppercase ‘Q’ indicates ‘QTL’; ‘kl’ represents kernel length, the abbreviation of the trait in this study; ‘BLE18’ indicates the mapping population; ‘4A’, represents the chromosome of this QTL.

### Validation of the major QTL

After obtaining the initial result of QTL mapping, the flanking markers were used to verify the effect of this QTL in another genetic background to further determine its stability, authenticity, and reliability. Based on the marker profiles, the population was divided into two categories: lines with homozygous alleles from either parent BLS1 or Sumai3 (excluding heterozygous lines). Then student’s *t*-test (*P* < 0.05) was utilized to detect significant difference between phenotypic data of two different types of lines.

### Comparison of QTL for KL on chromosome 4A

To confirm whether the major QTL detected in this study is a novel locus, the sequences of flanking KASP markers (*KASP-AX-111536305* and *KASP-AX-110174441*) for *Qkl.sicau-BLE18-4A* and those for previously identified QTL were utilized to perform blast searches against the reference genome sequence of *T. aestivum* cv. Chinese Spring (IWGSC RefSeq v2.1). Then, we compared the physical intervals to determine whether they overlap.

### Orthologous alignment

The flanking markers of the major QTL in this study were further aligned with the physical map of Chinese Spring (IWGSC RefSeq v2.1) and wild emmer (*T. turgidum* ssp *dicoccoides*, WEWSeq v2.0) to get orthologous genes. These genes were further analyzed for gene annotation and function on UniProt (http://www.uniprot.org/) [28, 31].

Furthermore, to identify the possible candidate genes of KL, the relative expression levels of the genes identified in the interval of *Qkl.sicau-BLE18-4A* were analyzed on the Triticeae Multiomics Center website (http://202.194.139.32/expression/wheat.html) and obtained through the wheat expression database of Chinese Spring cv-1 Development (single) [32].

## Results

### Phenotypic assessment and correlation analysis

**Fig. 2:**
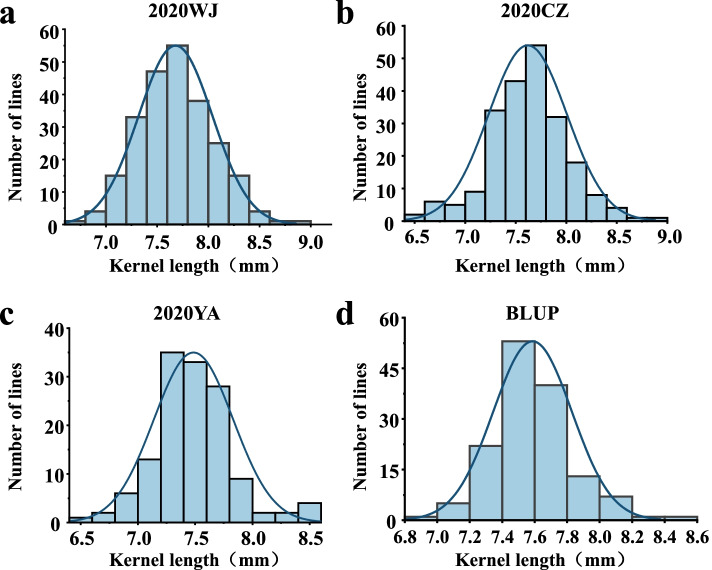
Frequency distributions for kernel length (KL) in BLE18 across different environments. Environment was defined with year and location. WJ, Wenjiang; CZ, Chongzhou; YA, Ya’an

Descriptive statistics for KL, KW and TKW of parents and two populations are presented in Table 1. Compared with BLS1, the KL values of 99E18 were significantly lower, but the KW values were significantly higher (Table 1and Fig. 1a). The KL and TKW values of BLS1 were significantly higher than Sumai3, except for individual environment (Table 1). The frequency distributions of KL showed approximate normal distributions in BLE18 across different environments (Fig. 2). The ranges of KL, KW and TKW were 6.53—8.98 mm, 3.17—4.29 mm, and 29.70 – 75.60 g, respectively, in BLE18 population. In all environments of BLSM3, KL ranged from 6.65 to 9.51 mm, KW from 2.68 to 4.24 mm and TKW from 34.57 to 67.40 g (Table 1). Both cell length and width of the kernel pericarp were significantly greater in BLS1 than in 99E18 and Sumai3, in agreement with the relationship of KL among three parents (Fig. 1b, c, d). The positive correlations for KL were detected among three environments with coefficient ranging from 0.52 to 0.55 in BLE18, and 0.43 to 0.56 among three environments in BLSM3 (*P* < 0.01, Table S3). The correlations among the three kernel traits were all significant for each other in BLE18 across multiple environments, with coefficient ranging from 0.17 to 0.79 (*P* < 0.05, Table 2). Significant and positive correlations were observed among KL, KW and TKW in BLSM3 across three environments, with correlation ranging from 0.03 to 0.76. Especially, there were extremely significant correlations (*P* < 0.01) between TKW and KL or KW in three environments, and the correlation coefficients ranged from 0.38 to 0.76 (Table 2).

**Table 1:** Phenotype of the parents and two populations in different environments

Trait	Environment	Parents		F_3_ population			
		P_1_	P_2_	Range	Mean	SD	CV (%)
BLE18 – BLS1 (P_1_) × 99E18 (P_2_)							
Kernel length (mm)	2020-WJ	8.63**	6.93	6.68–8.98	7.68	0.36	4.7
	2020-CZ	8.63**	6.91	6.59–8.83	7.63	0.41	5.33
	2020-YA	8.25**	6.53	6.53–8.52	7.49	0.39	5.16
	BLUP	8.50	6.98	6.98–8.50	7.60	0.23	3.05
Kernel width (mm)	2020-WJ	3.93**	4.07	3.54–4.29	3.96	0.13	3.16
	2020-CZ	3.64**	3.76	3.29–4.26	3.87	0.17	4.39
	2020-YA	3.47**	3.76	3.17–4.17	3.75	0.17	4.62
	BLUP	3.79	3.86	3.76–3.93	3.85	0.04	0.91
Thousand -kernel weight (g)	2020-WJ	62.05	57.57	42.47–74.33	59.99	5.04	0.08
	2020-CZ	60.67**	44.67	29.70–75.60	57.25	6.69	0.12
	2020-YA	/	43.00	32.00–74.00	50.12	7.11	0.14
	BLUP	57.10	51.77	49.04–63.72	56.08	2.42	0.04
BLSM3 – BLS1 (P_1_) × Sumai 3 (P_2_)							
Kernel length (mm)	2020-WJ	8.99**	6.44	6.65–9.51	8.03	0.48	5.98
	2020-CZ	9.09**	6.92	7.26–9.25	8.16	0.51	6.25
	2020-YA	8.28**	7.05	6.83–8.63	7.88	0.39	4.95
	BLUP	8.82	7.07	7.29–8.98	8.19	0.31	3.79
Kernel width (mm)	2020-WJ	3.66	3.75	2.68–4.24	3.76	0.26	6.91
	2020-CZ	4.05	4.11	3.31–4.21	3.9	0.18	4.62
	2020-YA	3.77**	3.80	3.03–4.12	3.63	0.21	5.79
	BLUP	3.83	3.84	3.60–3.89	3.82	0.07	1.83
Thousand -kernel weight (g)	2020-WJ	56.23**	44.83	39.77–67.40	54.55	5.75	0.11
	2020-CZ	61.20**	44.90	40.17–65.97	54.15	5.71	0.11
	2020-YA	50.30	46.00	34.57–60.37	46.04	5.86	0.13
	BLUP	53.81	48.19	46.53–56.13	51.48	1.81	0.04

Environment was defined with year and location. WJ, Wenjiang; CZ, Chongzhou; YA, Ya’an; BLUP, the best linear unbiased prediction; SD, standard deviation; CV, variation coefficient; /, data missing; **Significant at *P* < 0.01 between the two parents

**Table 2:** Correlation of different kernel traits in BLE18 and BLSM3 populations across different environments

Populations	Environments	Traits	KL	KW
BLE18	2020-WJ	KW	0.18**	1
		TKW	0.55**	0.56**
	2020-CZ	KW	0.17*	1
		TKW	0.49**	0.67**
	2020-YA	KW	0.25**	1
		TKW	0.46**	0.79**
BLSM3	2020-WJ	KW	0.10	1
		TKW	0.54**	0.54**
	2020-CZ	KW	0.03	1
		TKW	0.47**	0.44**
	2020-YA	KW	0.14	1
		TKW	0.38**	0.76**

*KL* kernel length (mm), *KW* kernel width (mm), *TKW*, thousand kernel weight (g), Environment was defined with year and location. WJ, Wenjiang; CZ, Chongzhou; YA, Ya’an; **Significant at P < 0.01, *Significant at *P* < 0.05

### BSA and Wheat 660 K analysis

**Fig. 3:**
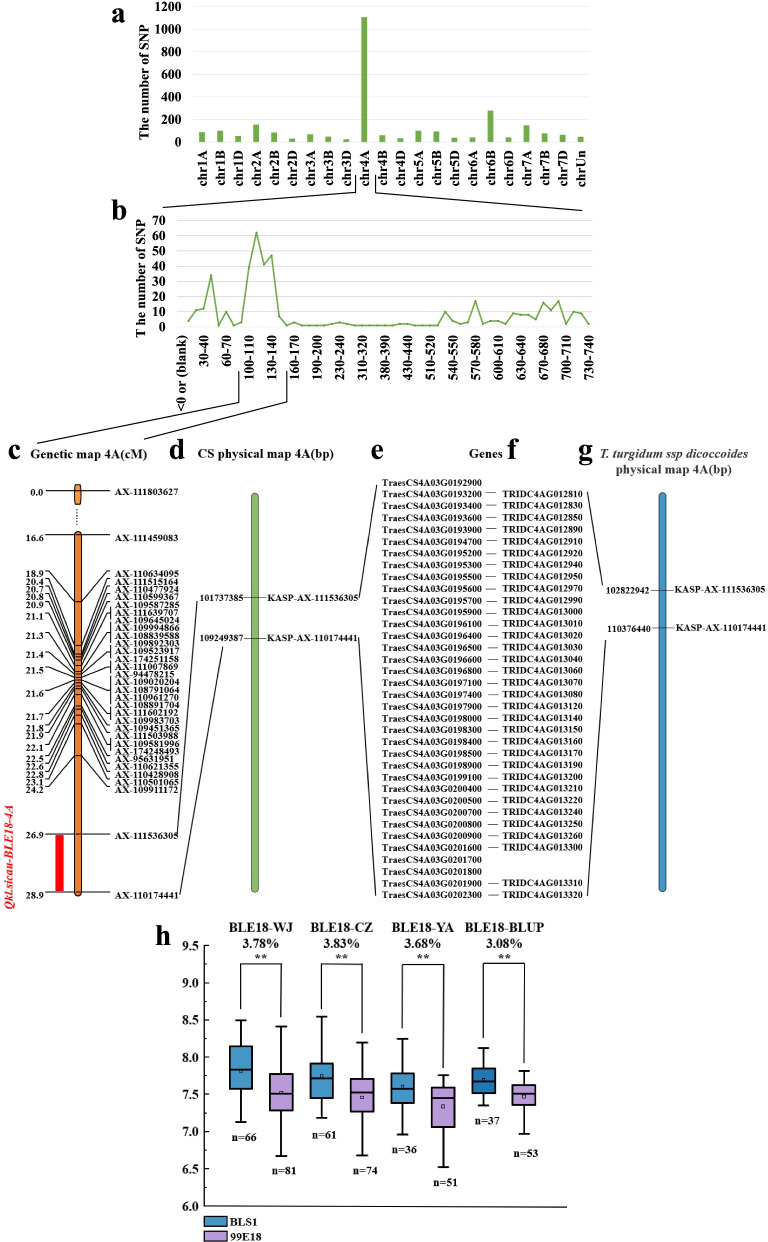
The results of genetic map and the effect of the major QTL for KL in BLE18 population. **a** The distribution of differential SNPs on chromosome in the mixing pool, transverse axis for different chromosomes, longitudinal axis for the number of SNP; **b** Distribution of different SNPs on chromosome 4A in different segments, transverse axis for chromosome position, longitudinal axis for the number of SNP; **c** The position of the markers on the genetic map; **d** The physical location of the flanking markers of *Qkl.sicau-BLE18—4A* in Chinese Spring; **e** Genes in the target region of the Chinese Spring physical map; **f** Genes homologous to Chinese Spring in the target region of the physical map of wild emmer wheat; **g** Physical location of flanking markers in wild emmer wheat; **h** The effect of the major QTL for KL in BLE18 population; WJ, Wenjiang; CZ, Chongzhou; YA, Ya’an; **Significance at the 0.01 probability level

After genotyping with the wheat 660 K SNP array, the number of homozygous polymorphic SNPs between the extreme pools and two parent pools were confirmed, resulting in a total of 764 SNPs, and 446 of them (58.4%) were observed on chromosome 4A (Fig. 3a). Most of the SNPs on 4A were within an interval of 90—160 Mb (Fig. 3b). The number of other SNPs unequally distributed across other chromosomes were ranging from 2 to 77. These results preliminarily determined that a locus controlling KL was most likely located on chromosome 4A.

### Genetic map construction and QTL mapping

A total of 33 KASP markers were developed based on polymorphic SNPs between two parents and two phenotypically contrasting pools on chromosome 4A, and were further used to genotype the population BLE18. Combined with the genotyping results of different markers, the genetic linkage map was constructed with a length of 28.9 cM (Fig. 3c).

A stable QTL (*Qkl.sicau-BLE18-4A*) for KL was detected in three environments as well as BLUP dataset. It was mapped between *KASP-AX-111536305* and *KASP-AX-110174441*, with an interval of 2 cM (Table 3 and Fig. 3c). It explained 10.87—19.30% of the phenotypic variation, with LOD values ranging from 3.01 to 6.41. The positive allele of *Qkl.sicau-BLE18-4A* was contributed by the parent BLS1, and the KL values of the lines carrying BLS1 alleles were significantly higher than those carrying 99E18 ones (Fig. 3h). No QTL for KW or TKW was identified on this chromosome. Further analysis also showed that this major QTL had no direct effect on KW and TKW (Figure S2).

**Table 3:** QTL (*Qkl.sicau-BLE18-4A*) for KL identified from different environments in BLE18 population

Environments	Chromosome	Left markers	Right markers	LOD	PVE	Add	Left CI	Right CI
2020-WJ	4A	*KASP-AX-111536305*	*KASP-AX-110174441*	6.41	14.19%	0.14	27.50	28.00
2020-CZ	4A	*KASP-AX-111536305*	*KASP-AX-110174441*	5.03	10.87%	0.15	26.50	28.00
2020-YA	4A	*KASP-AX-111536305*	*KASP-AX-110174441*	3.01	13.74%	0.12	27.50	28.00
BLUP	4A	*KASP-AX-111536305*	*KASP-AX-110174441*	5.79	19.30%	0.11	27.50	28.00

*LOD* logarithm of odds, *PVE* Percentage Variation Explained, *Add* additive effect of a QTL, *BLUP* best linear unbiased prediction, Environment was defined with year and location. WJ, Wenjiang; CZ, Chongzhou; YA, Ya’an; CI: confidence interval

After QTL × environment (QE) interaction analysis (Table S4), two QTL were detected, of which only one was the same as *Qkl.sicau-BLE18-4A*, and had higher LOD (19.16) and PVE values (12.19%), further indicating that *Qkl.sicau-BLE18-4A* was a major and stable QTL.

### Validation of the major QTL for KL

**Fig. 4:**
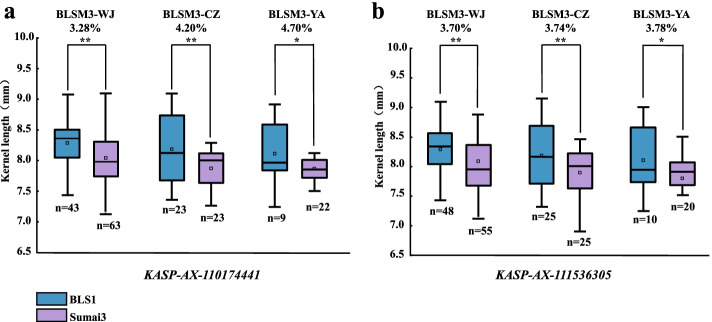
Effects of major QTL for KL in BLSM3 population across different environments. WJ, Wenjiang; CZ, Chongzhou; YA, Ya’an; **Significance at the 0.01 probability level; *significance at the 0.05 probability level

The flanking markers tightly linked to *Qkl.sicau-BLE18-4A* were used to verify the effects of *Qkl.sicau-BLE18-4A* in BLSM3 across different environments. The polymorphism between BLS1 and Sumai3 was detected using *KASP-AX-111536305* and *KASP-AX-110174441*. According to the genotyping results, the population were divided into two categories. The KL of the lines carrying positive alleles from BLS1 was significantly higher than that of the lines without positive alleles (*P* < 0.05, Fig. 4), and the differences between the two categories ranged from 3.28% to 4.70% in the validation population across three environments.

The homozygous lines of parental alleles ‘BLS1’ and ʻSuami3’ at *Qkl.sicau-BLE18-4A* were selected in accordance with the genotyping data of *KASP-AX-111536305* and *KASP-AX-110174441* for BLSM3 population. *T*-test showed that the KW and TKW of the lines carrying the ‘BLS1’ alleles were not significantly different from those of the lines without positive alleles in BLSM3 population across different environments, which further confirmed that *Qkl.sicau-BLE18-4A* had no genetic effect on KW and TKW (Figure S3).

## Discussion

### Qkl.sicau-BLE18-4A is a novel and stable QTL

Here, a major and stably expressed QTL, *Qkl.sicau-BLE18-4A* related to KL was detected in wheat between *KASP-AX-111536305* and *KASP-AX-110174441* on chromosome arm 4AS. In addition, its genetic effect was further validated in another verification population (Fig. 4), which suggested its reliability and stability.

*Qkl.sicau-BLE18-4A* was physically located between 101.74 Mbp and 109.25 Mbp on 4AS of Chinese Spring. To the best of our knowledge, only a few reports have documented QTL related to KL on 4A, and most of them were located on the long arm of 4A (4AL), such as *QKl-4A.1*, *qKL-4A*, *QKl.sdau-4A*, *QKL.sicau-4A*, *QGl.ccsu-4A.1* and *QKl.sau-4A.1* [11, 15, 33–38] (Table S5). In addition to the QTL mentioned above, we found some QTL on 4AS in previous reports. For example, *Q37*, a minor QTL, was located in the physical position of 47 Mbp, and the flanking marker *Xgwm397* of *Q37* was located at 47.3 Mbp [39]. *QGl-4A* was in the physical interval from 120.0 to 140.4 Mbp on 4AS and mapped into a 0.37 cM genetic interval [40]. *QKl.sau-4A.2* was co-located between 41.7 and 60.3 Mbp on chromosome 4AS of CS reference genome [38]. None of the above three genes overlapped with the interval of *Qkl.sicau-BLE18-4A*. In addition, as BLS1 is a mutant from an EMS-induced wheat genotype that was derived from the hybrid progeny of a spelt wheat and a common wheat. We think *Qkl.sicau-BLE18-4A* from this mutant may be different from previously reported ones, and should be most likely a novel locus. Further, spelt wheat has high nutritional value and contains all the basic components which are necessary for human beings including protein content and composition, lipids, crude fiber and vitamins [3]. Hybrids of common wheat and spelt wheat provide a positive selection by eliminating undesirable quality and improving nutritional value of bread wheat [41]. Thus, the lines carrying positive allele at *Qkl.sicau-BLE18-4A* should have breeding value given its spelt wheat genetic background.

### Analysis of the predicated genes in the interval of Qkl.sicau-BLE18-4A

We attempted to predict candidate genes for the identified QTL for KL on 4AS chromosome on the basis of the homology comparison results for Chinese Spring (IWGSC RefSeq v2.1) with wild emmer (WEWSeq v2.0) reference genomes. The physical location of *Qkl.sicau-BLE18-4A* is between 101.74 to 109.25 Mbp on Chinese Spring and 102.82 to 110.38 Mbp on wild emmer (Fig. 3 d, g). Searching analysis indicated that there were 36 genes in the physical map of Chinese Spring and 38 genes in the physical map of wild emmer wheat, with 33 in common (Table S6 and Fig. 3e, f).

For 33 orthologs, three previously reported genes might be related to kernel traits, and their relative expression levels (http://202.194.139.32/expression/wheat.html) were relatively high in kernels (Figure S4). For example, *TraesCS4A02G094300* encodes phosphatase 2C, a negative regulator of abscisic acid signaling [42]. *TraesCS4A02G094500* encodes tropinone reductases which function at the branch point of tropane alkaloid metabolism [43]. *TraesCS4A02G095500* encodes an adenyl-nucleotide exchange factor activity that was related to signal transmission [44].

Interestingly, the relative expression levels of *TraesCS4A03G0195700* and *TraesCS4A03G0196500* were the highest in the kernel compared with other tissues (leaves, spike and root). The expression levels of *TraesCS4A03G0193400* and *TraesCS4A03G0193900* in kernel were not the highest, but their expression levels in kernels were almost the same as other tissues (Figure S4). In summary, the above four genes are the emphasis in our subsequent research of fine mapping and gene cloning.

### Correlation between kernel traits

In this study, KL, KW and TKW were positively correlated in BLE18 across three environments. Notably, the correlation between KL and TKW, as well as between KW and TKW were positively significant (Table 2), which was consistent with previous studies [15, 34, 45–47].

Previous studies reported numerous co-located QTL for KL, KW and TKW. For example, *QKL.sicau-2D* for KL, *QKW.sicau-2D* for KW and *QTKW.sicau-2D* for TKW were co-located in the same interval. *QKL.sicau-1A* for KL and *QTKW.sicau-1A* for TKW were co-located, *QKL.sicau-2B* for KL and *QTKW.sicau-2B* for TKW were also co-located [15]. *TaTKW-7AL* for both TKW and KL were co-located on chromosome 7AL [13]. Interestingly, *Qkl.sicau-BLE18-4A* had no genetic effect on KW and TKW (Figure S2 and Figure S3), and the QTL for KW and TKW were not identified in the same interval, which indicated that the QTL for KL may have different regulation mechanism in this study. Recently, Qu et al. (2021) also identified major QTL *QKL.sicau-2SY-1B* for KL, *QKW.sicau-2SY-6D* for KW, and *QTKW.sicau-2SY-2D* for TKW located on different chromosomes [16].

Additionally, we should notice that when analyzing effects of a QTL for a given trait on other traits, loci of these corresponding traits in lines harboring the given QTL should be excluded. Otherwise, real effect of a given QTL may be covered and cannot be detected. In our present study, only one major QTL for KL (*Qkl.sicau-BLE18-4A*) was detected. We also did not detect any QTL for KW and TKW in this interval, suggesting that loci for these two traits may be located in other intervals or chromosomes. Therefore, in our analysis of effects of *Qkl.sicau-BLE18-4A* on KW and TKW, the loci for KW and TKW mapped on other intervals may interfere the detection ability, resulting in no significant difference in KW or TKW between lines with the allele from BLS1 and that from 99E18 for *Qkl.sicau-BLE18-4A.* We thus suggest that genome-wide analysis of loci for KL, KW and TKW could factually reveal their genetic correlations. Anyway, the major and novel QTL for KL identified in this study could be useful for dissecting mechanism of KL in the future and could have potential in breeding. Aggregating *Qkl.sicau-BLE18-4A* with other loci/genes controlling KW may have significant effects on TKW.

The kernels are developed from the carpel during wheat flowering, and then the endosperm cells begin to divide and expand, following by the accumulation of starch and protein. KL and KW determined the kernel morphology. Larger kernel size was related to higher kernel weight [48].Thus, among many factors that constitute kernel weight, KL and KW play a vital role that cannot be ignored.

## The feasibility of QTL mapping of quantitative traits using BSA and wheat 660 K SNP arrays in low generation populations

BSA has been widely used in wheat genetic mapping, and it is one of the effective methods to rapidly obtain target genes [18, 49, 50]. For species with large and complex genomes like wheat, it is a huge and laborious project to complete genome sequencing. Therefore, more convenient, faster and accurate methods are needed to improve the credibility of the results in mapping of wheat traits. So far, many studies have been using BSA combined with various sequencing methods to achieve gene localization [51, 52]. For example, the combination of BSA and the wheat 660 K SNP array has been widely used for localization of interest genes especially for disease resistant loci. A major QTL (*Qyrnap.nwafu-2BS*) for stripe rust resistance was identified on chromosome arm 2BS following BSA and the wheat 660 K SNP array analysis in an F_2:3_ population [19]. The early leaf senescence gene *Els2* was mapped in an F_2_ population using BSA and the wheat 660 K SNP arrays [53]. However, a few studies had shown that BSA—660 K SNP arrays can be used to identify QTL for agronomic traits in low generations of wheat. Recently, a total of 48 QTL controlling spike-related traits located on 18 chromosomes were identified using BSA and the wheat 660 K SNP array in genetic analysis of F_2_ and F_2:3_ populations [40]. Another example is that a novel reduced height gene was localized on chromosome arm 2DL using BSA—660 K SNP arrays in four F_2_ segregating populations [54]. In this study, two low-generation (F_3_) populations were used to rapidly identify and validate QTL related to KL. This method greatly improved the efficiency and shortened the time. Collectively, our results combined with previous studies suggested that QTL mapping of agronomic traits using BSA and the wheat 660 K SNP array in combination with linkage map analysis in low generation populations is a rapid and effective method.

## Conclusion

A major, novel and stable QTL for KL, *Qkl.sicau-BLE18-4A*, was identified in an F_3_ biparental population derived from the cross between BLS1 and 99E18, and further successfully validated using two flanking makers in another F_3_ biparental population across different environments. Four predicted genes related to kernel growth regulation were detected in the interval of *Qkl.sicau-BLE18-4A*. Furthermore, this study laid subsequent construction for fine mapping, mining candidate genes and researching on the molecular mechanism of KL regulation in the future.

## Supplementary Information

**Additional file 1: Figure S1.** Kernel phenotypes of BLS1 and Sumai3.Scale bar = 1cm. **Table S1.** The statistics of linesused and not used for trait investigation in BLE18 in Ya’an. **Table S2.** The primers used in this study. **Table S3. **Correlation of KL between differentenvironments in BLE18 and BLSM3 populations. **Figure S2. **Effect of *Qkl.sicau-BLE18-4A*on kernel width **a** and thousand kernel weight **b** in BLE18 population. WJ, Wenjiang;CZ, Chongzhou; YA, Ya’an. **Table S4. **QTLfor KL detected in QTL-environment (QE) interaction analysis. **Figure S3. **Effect of *Qkl.sicau-BLE18-4A* on kernel widthand thousand kernel weight in BLSM3 population. WJ, Wenjiang; CZ, Chongzhou; YA, Ya’an. **Table  S5. **QTL for KL identified on 4A chromosome fromprevious studies. *Bold font*, QTL obtained in this experiment; N,information could not be found. **Table S6. **Gene annotation of the major QTL (*Qkl.sicau-BLE18-4A*) interval onChinese Spring physical map. **Figure S4.** The relative expression levelsof genes possibly related to kernel development in different organs. Transverseaxis is different parts of plants, from left to right are roots, leaves andkernels. Transverse axis is the expression level.

## Data Availability

The data that support the findings of this study are available in ‘Figshare’ with the identifiers data DOIs, including dataset 1 (the genotypes of BLE18 population, https://doi.org/10.6084/m9.figshare.19514173.v1), dataset 2 (the genetic map information in this study, https://doi.org/10.6084/m9.figshare.19514239.v1), dataset 3 (data of kernel traits of BLE18 in three environments, https://doi.org/10.6084/m9.figshare.19514248.v1), dataset 4 (data of kernel traits of BLSM3 in three environments, https://doi.org/10.6084/m9.figshare.19514254.v1) and dataset 5 (the genotyping results of two flanking markers of *Qkl.sicau-BLE18-4A* in BLSM3, https://doi.org/10.6084/m9.figshare.19514212.v1). Remaining data generated or analyzed during this study are included in this article and its additional files.

## Abbreviations

BSA: Bulked Segregant Analysis
SNP: A single nucleotide polymorphism
BLUP: Best linear unbiased prediction
QTL: Quantitative trait loci
KL: Kernel length
KT: Kernel thickness
KW: Kernel width
HKW: Hundred kernel weight
TKW: Thousand kernel weight
AFLP: Amplified fragment length polymorphism
SSR: Simple sequence repeats
KASP: Kompetitive Allele Specific PCR
PVE: Phenotypic variance explained
LOD: Logarithm of odds
Add: Additive effect of a QTL
QE: QTL × Environment
CS: Chinese Spring
CZ: Chongzhou
WJ: Wenjiang
YA: Ya’an
