# Supplementary material for: Identification and validation of a major QTL for kernel length in bread wheat based on two F3 biparental populations
Source: BMC Genomics. 2022 May 19;23:386. doi: 10.1186/s12864-022-08608-3 (PMC9121568; doi:10.1186/s12864-022-08608-3)
Supplement: Supplementary file 1 — Additional file 1: Figure S1. Kernel phenotypes of BLS1 and Sumai3.Scale bar = 1cm. Table S1. The statistics of linesused and not used for trait investigation in BLE18 in Ya’an. Table S2. The primers used in this study. Table S3. Correlation of KL between differentenvironments in BLE18 and BLSM3 populations. Figure S2. Effect of Qkl.sicau-BLE18-4Aon kernel width a and thousand kernel weight b in BLE18 population. WJ, Wenjiang;CZ, Chongzhou; YA, Ya’an. Table S4. QTLfor KL detected in QTL-environment (QE) interaction analysis. Figure S3. Effect of Qkl.sicau-BLE18-4A on kernel widthand thousand kernel weight in BLSM3 population. WJ, Wenjiang; CZ, Chongzhou; YA, Ya’an. Table S5. QTL for KL identified on 4A chromosome fromprevious studies. Bold font, QTL obtained in this experiment; N,information could not be found. Table S6. Gene annotation of the major QTL (Qkl.sicau-BLE18-4A) interval onChinese Spring physical map. Figure S4. The relative expression levelsof genes possibly related to kernel development in different organs. Transverseaxis is different parts of plants, from left to right are roots, leaves andkernels. Transverse axis is the expression level. [file 12864_2022_8608_MOESM1_ESM.docx]

**Identification and validation of a major QTL for kernel length in bread wheat based on two F_3_ biparental populations**

Xinlin Xie^2,^ ^#^, Shuiqin Li^2, #^, Hang Liu^2^, Qiang Xu^1^, Huaping Tang^1^, Yang Mu^1^, Mei Deng^1^, Qiantao Jiang^1^, Guoyue Chen^1^, Pengfei Qi^1^, Wei Li^3^, Zhien Pu^3^, Ahsan Habib^4^, Yuming Wei^1^, Youliang Zheng^1^, Xiujin Lan^1, *^, Jian Ma^1, *^

*^1^*State Key Laboratory of Crop Gene Exploration and Utilization in Southwest China, Sichuan Agricultural University, Chengdu, 611130, China

*^2^*Triticeae Research Institute, Sichuan Agricultural University, Chengdu, 611130, China

*^3^*College of Agronomy, Sichuan Agricultural University, Chengdu, 611130, China

*^4^*Biotechnology and Genetic Engineering Discipline, Khulna University, Khulna 9208, Bangladesh

# Contributed equally to this paper.

*Authors for correspondence:

Dr. Jian Ma, E-mail: jianma@sicau.edu.cn

Dr. Xiujin Lan, E-mail: lanxiujin@163.com

Tel: +86 28 86293115; Fax: +86 28 82650350

The supplementary information is presented in the form of a single file including PDF format and XLSX format, rather than being concentrated in this DOCX document.

**Additional files description**

**Additional file 1: Figure S1.** Kernel phenotypes of BLS1 and Sumai3. Scale bar = 1 cm.

**Additional file 2: Table S1.** The statistics of lines used and not used for trait investigation in BLE18 in Ya’an.

**Additional file 3: Table S2.** The primers used in this study.

**Additional file 4: Table S3.** Correlation of KL between different environments in BLE18 and BLSM3 populations.

**Additional file 5: Figure S2.** Effect of *Qkl.sicau-BLE18-4A* on kernel width (**a**) and thousand kernel weight (**b**) in BLE18 population. WJ, Wenjiang; CZ, Chongzhou; YA, Ya’an.

**Additional file 6: Table S4.** QTL for KL detected in QTL-environment (QE) interaction analysis.

**Additional file 7: Figure S3.** Effect of *Qkl.sicau-BLE18-4A* on kernel width and thousand kernel weight in BLSM3 population. WJ, Wenjiang; CZ, Chongzhou; YA, Ya’an.

**Additional file 8: Table S5.** QTL for KL identified on 4A chromosome from previous studies. *Bold font*, QTL obtained in this experiment; N, information could not be found.

**Additional file 9: Table S6.** Gene annotation of the major QTL (*Qkl.sicau-BLE18-4A*) interval on Chinese Spring physical map.

**Additional file 10: Figure S4.** The relative expression levels of genes possibly related to kernel development in different organs. Transverse axis is different parts of plants, from left to right are roots, leaves and kernels. Transverse axis is the expression level.
